# Long-Term Efficacy and Safety of Leuprorelin Treatment in Children with Central Precocious Puberty: A Systematic Review and Meta-Analysis

**DOI:** 10.3390/children12060712

**Published:** 2025-05-30

**Authors:** Ling Hou, Yanqin Ying, Feng Ye, Cai Zhang, Xiaoping Luo

**Affiliations:** Department of Pediatrics, Tongji Hospital of Tongji Medical College, Huazhong University of Science and Technology, Wuhan 430030, China; linghou@tjh.tjmu.edu.cn (L.H.); yqying@tjh.tjmu.edu.cn (Y.Y.); doctoryf@126.com (F.Y.); caizhang@tjh.tjmu.edu.cn (C.Z.)

**Keywords:** leuprorelin, central precocious puberty, children, long-term, meta-analysis

## Abstract

**Background:** As the first approved GnRH agonist, leuprorelin is distinguished by its broad application in managing central precocious puberty (CPP). Despite the extensive use of leuprorelin in CPP management, uncertainties still persist regarding its long-term efficacy and safety. We conducted a systematic review and meta-analysis to assess the long-term efficacy and safety of leuprorelin treatment in children with CPP. **Methods:** We conducted electronic searches in PubMed, Embase, and the Cochrane Library up until 15 November 2023. All relevant studies concerning leuprorelin treatment in children with CPP were included. **Results:** The final adult height of children with CPP eventually reached the target height, with a significant difference of MD: 1.75 cm (95% CI: 0.46–3.03). The MD in BMI standard deviation score between baseline and post-leuprorelin treatment was −0.03 (95% CI: −0.28–0.22). For the onset of menstrual puberty, the MD between children with CPP who received leuprorelin treatment and those who did not was 0.73 years latency (95% CI: −0.74–2.20) without significant difference. The timing of menstrual puberty of the leuprorelin-treated group was 15.83 months (95% CI: 11.62–20.03) after the discontinuation of leuprorelin treatment. The proportion of menstrual regularity was 85% (95% CI: 75–91%), and the average incidence rate of polycystic ovary syndrome (PCOS) was 8% (95% CI: 3–22%) for children with CPP that treated with leuprorelin. **Conclusions:** Leuprorelin treatment does not affect BMI or the onset of menstrual puberty in the long term, but has positive effects on adult height for children with CPP. Moreover, no severe adverse events related to leuprorelin treatment were observed.

## 1. Introduction

Central precocious puberty (CPP) is characterized by the premature activation of the hypothalamic–pituitary–gonadal axis, resulting in premature pubertal development. This includes breast development and menarche occurring before the ages of 8 and 10, respectively, in girls, while testicular enlargement occurs before the age of 9 in boys [1,2,3]. Menarche before the age of 10 should be carefully evaluated to confirm CPP and exclude other underlying conditions. CPP is significantly more prevalent in girls, with an incidence up to 15 times higher in girls than in boys [4]. Beyond its physical manifestations, CPP has profound psychological and social implications on affected children, including reduced adult height, emotional distress, and potential engagement in risky behaviors [2].

To improve CPP in children, Gonadotrophin-releasing hormone analogs (GnRHa) have been the gold-standard treatment since the mid-1980s [5]. These medications aim to suppress gonadotropin secretion, delay puberty progression, slow bone maturation, and improve adult height outcomes [4,5]. Among GnRHa, leuprorelin has emerged as a widely used option due to its effectiveness in halting pubertal development and its favorable safety profile [4,5]. Leuprorelin, a synthetic analog of GnRH, operates by desensitizing the pituitary gland to GnRH, thereby reducing the secretion of luteinizing hormone (LH) and follicle-stimulating hormone (FSH), which are responsible for gonadal steroidogenesis [6].

As the first approved GnRH agonist, leuprorelin stands out for its extensive use in managing CPP. Despite its widespread application, no meta-analysis has specifically evaluated its long-term efficacy, including effects on reproductive function, growth and development, or its safety profile, leaving uncertainties about critical outcomes such as bone density and polycystic ovary syndrome [6]. Previous meta-analyses have indicated the potential of GnRHa, including leuprorelin, to improve final adult height and decrease body mass index (BMI) in girls with idiopathic CPP [7]. Additionally, various consensus statements and recommendations have outlined the available GnRHa and therapeutic regimens for CPP treatment [2,3,8]. However, these resources have not exclusively focused on leuprorelin treatment for children with CPP. Moreover, many prior studies have been limited by small sample sizes, which hinders the reliability and generalizability of their conclusions. To deepen our understanding of leuprorelin’s benefits in CPP management, we conducted a systematic review and meta-analysis to evaluate the long-term efficacy and safety of leuprorelin treatment in affected children. Our analysis involved comparing leuprorelin usage to non-usage or pre- and post-leuprorelin treatment scenarios.

## 2. Materials and Methods

This systematic review and meta-analysis strictly follows the methods outlined in the Cochrane Handbook [9] and adheres to PRISMA reporting guidelines [10]. The protocol was registered on the PROSPERO: CRD42024586307.

### 2.1. Study Searches and Selection

We conducted a comprehensive search for relevant studies up to 15 November 2023. Our electronic searches covered three databases: PubMed, Embase, and the Cochrane Library. No language restrictions were applied. The search terms encompassed various aspects, including leuprorelin, central precocious puberty, clinical studies, and observational studies. Detailed information about these search terms can be found in Supplementary S1.

After removing duplicate records, two reviewers screened articles based on their titles and abstracts. Discrepancies that emerged were addressed through discussion and, if needed, resolved by a third reviewer. Our inclusion criteria encompassed the following: (1) single-arm studies and prospective or retrospective comparative studies; (2) children with CPP (as defined in the original studies); and (3) studies that reported long-term outcomes for children with CPP. We acknowledge the diagnostic challenges associated with CPP, given its broad spectrum encompassing isolated premature thelarche, constitutional growth acceleration, and both progressive and non-progressive forms. To mitigate potential biases, we included studies that adhered to established diagnostic criteria, such as clinical signs of puberty before age 8 in girls, advanced bone age, and elevated basal or stimulated luteinizing hormone (LH) levels. The definition of long-term is reaching adult height or final adult height after treatment, having follow-up after treatment cessation, or the onset or recurrence of menstruation. The exclusion criteria included reviews, letters, editorials, student theses, abstracts, case reports, case series, studies published before 2000, and studies without leuprorelin treatment.

The risk of bias was assessed using the Newcastle Ottawa Scale (NOS) [11], which employs a star scoring system with a maximum score of nine points. Studies that scored six or above were considered to be of high quality. Any discrepancies were resolved through discussion, with the involvement of a third reviewer for judgment if needed.

### 2.2. Outcomes

In this meta-analysis, we focus on three primary long-term outcomes: growth and development, reproductive function, and bone metabolism. Growth and development encompassed final adult height, adult height, and BMI. Reproductive function included aspects such as menstrual regularity and duration, the presence of polycystic ovary syndrome, and fertility (e.g., normal pregnancy). Bone metabolism was assessed through bone mineral density (BMD).

### 2.3. Statistical Analysis

In this study, we performed the meta-analyses of single-arm studies and comparative studies separately. For continuous outcomes, we calculated the mean difference (MD), corresponding 95% confidence intervals (CIs), and *p* value for each study. For dichotomous outcomes, we evaluated the risk ratio (RR) along with 95% CIs.

To account for variations between studies, we applied a random effects model to calculate the summary RR and MD for each outcome. Heterogeneity was assessed using the I^2^ statistic [12], with I^2^ values interpreted as follows: I^2^ ≤ 25% indicating no heterogeneity, 26% to 50% suggesting a low degree of heterogeneity, 51% to 75% indicating a moderate degree of heterogeneity, and ≥75% signifying a high degree of heterogeneity.

All statistical analyses were conducted using either R software (version 4.0.4) or Review Manager Software (RevMan version 5.4; The Nordic Cochrane Centre, The Cochrane Collaboration, Copenhagen, Denmark). Two-sided statistical tests were conducted with significance levels set at *p* < 0.05 unless specified otherwise.

## 3. Results

### 3.1. Overview of Studies Included in the Meta-Analysis

**Figure 1:**
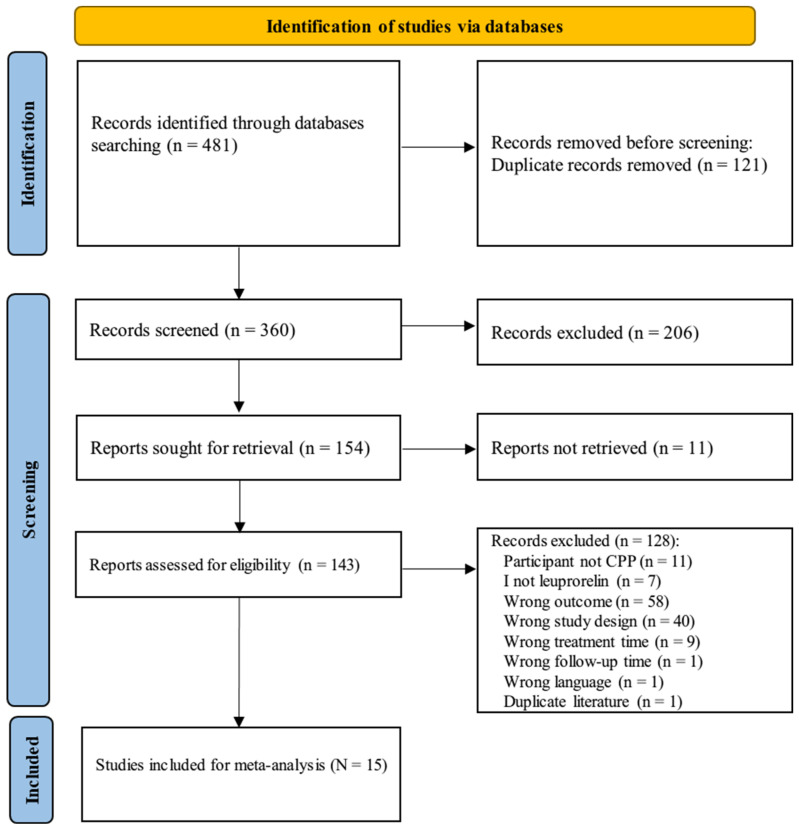
PRISMA flow chart for literature identification on leuprorelin treatment in children with central precocious puberty. Search terms are described in Supplementary S1.

Initially, 481 articles were identified. After removing duplicates, 360 articles remained, with 206 articles excluded. Following a full-text review of 143 articles, 15 were selected for inclusion in this meta-analysis (Figure 1). A summary of these studies is provided in Table 1.

Among the 15 included studies [13,14,15,16,17,18,19,20,21,22,23,24,25,26,27], the majority of patients were girls, accounting for 96% (Table 1). The average age of patients with CPP at the initiation of leuprorelin treatment ranged from 6.8 to 13.7 years old in girls and from 7.9 to 9.8 years old in boys. Treatment was consistently administered through intramuscular/subcutaneous injection. Nearly all studies reported on the height of children with CPP treatment. However, only two articles simultaneously reported on target height, final adult height, and lifetime height [24,25]. Various studies focused on different outcome measurements, with fewer reports on other long-term outcomes, ranging from one to three studies each. These studies, conducted between 2011 and 2022, collectively involved 795 children with CPP treated with leuprorelin.

**Table 1:** Baseline characteristics of studies included in the meta-analysis.

Number	Study ID	Study Design	Group	Sample Size (n)	Age (years) (Mean ± SD)	Male/Female n/n	Intervention	Usage and Dosage
1	Somchit Jaruratanasirikul 2011 [18]	Cohort study	Experimental group	32	6.8 ± 1.9	0/32	Leuprorelin	Intramuscular injection 90–140 μg/kg/4 weeks
			Control group	20	7.7 ± 1.9	0/20	Untreated (CPP)	Untreated
2	Vickie Wu 2021 [19]	Single-arm trial	Experimental group	18	9.7 ± 1.7	0/18	Leuprorelin	Intramuscular injection 15 mg/months or 30 mg/3 months
3	Sun-Jin Lee 2022 [20]	Single-arm trial	Experimental group	27	11.3 ± 0.5	0/27	Leuprorelin	Intramuscular injection 0.2–0.3 mg/kg/4 weeks
4	Shinyoung Jang 2022 [21]	Cohort study	Experimental group	72	8.2 ± 0.8	0/72	Leuprorelin	Intramuscular injection 3.75 mg/subcutaneous injection
			Control group	14	8.5 ± 0.6	0/14	Leuprorelin	Intramuscular injection 60–85 μg/kg/4 weeks
5	Renata Iannetta 2015 [15]	Cohort study	Experimental group	27	13.3 ± 1.3	0/27	Leuprorelin	Intramuscular injection 3.75 mg/month
			Control group	26	13.7 ± 1.4	0/26	Untreated (healthy population)	Untreated
6	Pınar Şimşek Onat 2020 [22]	Cohort study	Experimental group	19	7.3 ± 0.5	0/19	Leuprorelin	Intramuscular injection 3.75 mg/28 days
			Control group	35	9.0 ± 0.5	0/35	Leuprorelin	Intramuscular injection 3.75 mg/28 days
7	Peter A Lee 2011 [23]	Single-arm trial	Experimental group	49	7.3 ± 1.9	0/49	Leuprorelin	Intramuscular injection 3.75 mg/28 days
8	Hae Sang Lee 2018 [24]	Observational study (single arm)	Experimental group	84	8.2 ± 0.6	0/84	Leuprorelin	Weight > 30 kg: intramuscular injection 3.75 mg/28 days Weight 20–30 kg: intramuscular injection 2.5 mg/28 days Weight < 20 kg: intramuscular injection 1.87 mg/28 days
9	Carolina O. Ramos 2021 [17]	Single-arm trial	Experimental group	22	8.3 ± 0.9	0/22	Leuprorelin	Intramuscular/subcutaneous injection 11.25 mg/12 weeks
10	Ah Young Cho 2020 [25]	Cohort study	Experimental group	50	8.5 ± 0.8	0/50	Leuprorelin	Intramuscular injection 60 μg/28 days
			Control group	19	8.7 ± 0.8	0/19	Leuprorelin	Intramuscular injection 60 μg/28 days
11	Yi-Chun Lin 2017 [26]	Cohort study	Experimental group	43	8.34 ± 1.2	0/43	Leuprorelin	Intramuscular injection 3.75 mg/month
			Control group	44	9.5 ± 1.0		Leuprorelin	Intramuscular injection 3.75 mg/month
12	Toshiaki Tanaka 2005 [13]	Controlled clinical trial	Experimental group	63	7.7 ± 2.2	0/63	Leuprorelin	Initial dose: Subcutaneous injection 10/30/90 g/kg/4 weeks
			Control group	13	9.8 ± 1.8	13/0	Leuprorelin	Initial dose: Subcutaneous injection 10/30/90 g/kg/4 weeks
13	Maria de Fátima Borges 2015 [27]	Single-arm trial	Experimental group	62	6.9 ± 1.7	8/54	Leuprorelin	Intramuscular injection 3.75 mg/months
14	Inge M. van der sluis 2002 [16]	Single-arm trial	Experimental group	47	8.3 (range, 2.8–11.4)	5/42	Leuprorelin	First month: Subcutaneous injection of 3.75 mg/2 weeks Routine: Subcutaneous injection of 3.75 mg/4 weeks
15	E. Kirk Neely 2010 [14]	Single-arm trial	Experimental group	49	7.3 ± 1.9	0/49	Leuprorelin	Intramuscular injection ≥ 300 µg/kg (7.5/11.25/15.0 mg)/28 days
			Experimental group	6	7.9 ± 2.0	6/0	Leuprorelin	Intramuscular injection ≥ 300 µg/kg (7.5/11.25/15.0 mg)/28 days

In this study, only one trial was a non-randomized controlled trial [13], while others were either single-arm or comparative studies. The data from the non-randomized controlled trial were treated as comparative due to the utilization of before and after treatment data from one group [13]. Consequently, all studies were assessed for quality using the NOS (Supplementary Table S1). While all studies achieved high NOS scores of 7–8 points, the risk of bias was low across all included studies.

### 3.2. Growth and Development

**Figure 2:**
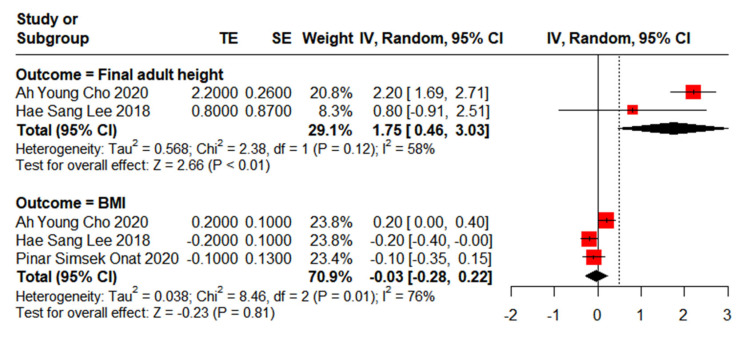
Meta-analysis of final adult height and BMI for children with central precocious puberty after leuprorelin treatment [22,24,25].

Adult height is often considered a crucial indicator of growth and development in children. In this meta-analysis, we identified two studies involving a total of 153 children with CPP to evaluate their final adult height following leuprorelin treatment (Figure 2). A moderate degree of heterogeneity was observed among these studies (I^2^ = 58%). Overall, after leuprorelin treatment, the final adult height eventually reached the target height, and with a significant difference of MD of 1.75 cm (95% CI: 0.46–3.03). These findings suggest that the use of leuprorelin positively impacts adult height attainment.

Regarding BMI, three studies were analysed, comprising a total of 207 children with CPP who underwent leuprorelin treatment (Figure 2). A high degree of heterogeneity was observed among these studies (I^2^ = 76%).

Overall, the mean difference in BMI standard deviation score (SDS) between baseline and post-leuprorelin treatment was −0.03 (95% CI: −0.28–0.22), indicating no significant effects of leuprorelin treatment on BMI.

### 3.3. Reproductive Function

**Figure 3:**
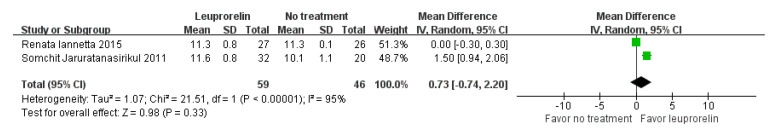
Meta-analysis of onset of menstrual puberty for children with central precocious puberty after leuprorelin treatment [15,18].

For the onset of menstrual puberty, two studies involving a total of 105 children with CPP who underwent leuprorelin treatment were analysed (Figure 3). Considerable heterogeneity was observed among these studies (I^2^ = 95%). Overall, the mean difference in the onset of menstrual puberty between children with CPP who received leuprorelin treatment and those who did not was 0.73 years (95% CI: −0.74–2.20), indicating no statistically significant difference in the onset of menstrual puberty.

**Figure 4:**
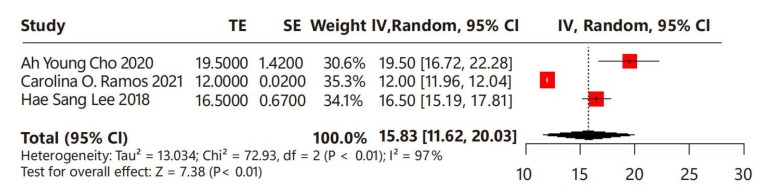
Meta-analysis of timing of menstrual puberty for children with central precocious puberty after the discontinuation of leuprorelin treatment [17,24,25].

Regarding the timing of menstrual puberty after leuprorelin treatment, three studies were examined, comprising a total of 175 children with CPP (Figure 4). A substantial degree of heterogeneity was noted among these studies (I^2^ = 97%). Overall, the timing of menstrual puberty of the leuprorelin-treated group was 15.83 months (95% CI: 11.62–20.03) after the discontinuation of leuprorelin treatment.

**Figure 5:**
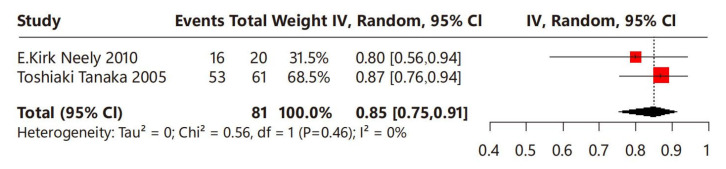
Meta-analysis of menstrual regularity for children with central precocious puberty after leuprorelin treatment [13,14].

In terms of menstrual regularity, two studies were included, involving a total of 81 children with CPP who underwent leuprorelin treatment (Figure 5). No heterogeneity was observed among these studies (I^2^ = 0%). Overall, the proportion of menstrual regularity of the leuprorelin-treated group was 85% (95% CI: 75–91%), suggesting that 85% of children with CPP achieved menstrual regularity after leuprorelin treatment.

Regarding fertility, only one study reported the fertility status of children with CPP that were followed into adulthood [15]. Among those with mature fertility, nine out of eleven females exhibited detectable ovulatory menstrual cycles, while all six boys had serum testosterone levels within the normal adult range.

**Figure 6:**
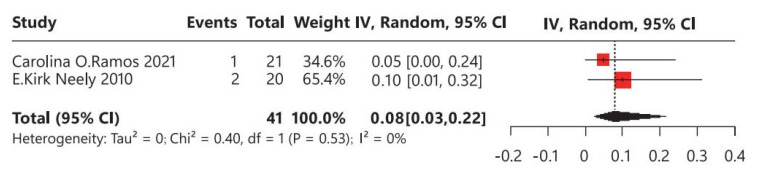
Meta-analysis of polycystic ovary syndrome for children with central precocious puberty after leuprorelin treatment [14,17].

In terms of polycystic ovary syndrome (PCOS), two studies were identified, involving a total of 41 children with CPP who underwent leuprorelin treatment (Figure 6). No heterogeneity was observed among these studies (I^2^ = 0%). Overall, the average incidence rate of PCOS was 8% (95% CI: 3–22%), indicating that approximately 8% of children with CPP may develop PCOS following leuprorelin treatment.

### 3.4. Bone Metabolism

For BMD, two studies involving a total of 71 children with CPP who underwent leuprorelin treatment were identified [15,16]. However, due to variations in data format, a meta-analysis could not be conducted (Supplementary Table S2). One study found no statistical difference in BMD before and after leuprorelin treatment [15]. Conversely, another study suggested an increase in BMD before 2 years but a potential decrease in BMD of the total femur after 2 years of treatment with leuprorelin [16]. Regarding BMD in the lumbar vertebrae, there was an increase observed following leuprorelin treatment compared with the baseline during a 3-year period.

## 4. Discussion

This systematic review and meta-analysis, comprising 15 studies and 795 patients, provides a comprehensive evaluation of the long-term efficacy and safety of leuprorelin treatment in children with CPP. Our findings contribute valuable evidence regarding the potential benefits of leuprorelin therapy in this population. Specifically, we observed that adult height was significant higher than the target height among children with CPP receiving leuprorelin treatment. However, in contrast to baseline conditions, leuprorelin treatment did not impact the BMI of affected children. Furthermore, our analysis revealed no statistically significant difference in the onset of menstrual puberty before and after leuprorelin treatment.

Our assessment extended to other relevant outcomes, including the timing of menstrual puberty and menstrual regularity. We found that the onset of menstrual puberty in children with CPP was 15.83 months after the discontinuation of leuprorelin treatment. Moreover, a majority of children with CPP achieved menstrual regularity after receiving leuprorelin treatment. These findings underscore the broad and positive impact of leuprorelin treatment on CPP management, potentially leading to improved patient outcomes.

The timing of menstrual puberty, occurring within a physiologically appropriate window after treatment cessation, suggests that leuprorelin effectively supports the resumption of normal pubertal development without undue delays. Furthermore, the achievement of menstrual regularity in most patients reflects the restoration of a balanced hypothalamic–pituitary–gonadal axis, which is critical for long-term reproductive health. These outcomes highlight not only the efficacy of leuprorelin in addressing the immediate challenges of CPP but also its potential role in ensuring favorable developmental trajectories. However, additional longitudinal studies are needed to evaluate how these benefits extend into adulthood, particularly regarding fertility and endocrine health.

In addition to efficacy, we thoroughly evaluated the safety profile of leuprorelin treatment. Our analysis revealed that children with CPP receiving leuprorelin treatment had an incidence rate of PCOS of approximately 8%, which falls slightly below the normal range of 8–13% for reproductive-age women [28]. Thus, leuprorelin treatment may offer a favorable safety profile for children with CPP. The observed incidence of PCOS in treated individuals, being slightly lower than the reported range for reproductive-age women, suggests that leuprorelin may not significantly increase the risk of this condition. Additionally, the data underline the importance of continuous monitoring and further research to confirm these trends over extended follow-up periods while investigating other potential long-term safety outcomes, such as BMD and metabolic health. However, limitations in our data prevented a comprehensive evaluation of leuprorelin’s impact on BMD in children with CPP. Previous evidence suggests that children undergoing GnRHa treatment, including leuprorelin, may experience a temporary decrease in BMD, a trend consistent with findings from one study in our meta-analysis [16]. This transient reduction in bone density is likely due to the suppression of gonadal hormones, which play a critical role in bone mineralization during puberty. However, as GnRHa therapy is discontinued and normal pubertal progression resumes, BMD typically recovers to normal levels following the cessation of therapy [3], suggesting that leuprorelin treatment may minimize its adverse effects on BMD in adulthood for children with CPP. Long-term follow-up studies are essential to confirm whether bone density fully normalizes and to assess whether any residual deficits persist into adulthood.

To the best of our knowledge, this is the first systematic review and meta-analysis to evaluate the long-term efficacy and safety of leuprorelin treatment in children with CPP. Previous studies have primarily been limited by small sample sizes or narrative reviews [29]. By synthesizing data from 15 studies involving 795 patients, our analysis provides exhaustive insights into the effects of leuprorelin treatment on various outcomes in children with CPP. These insights hold significant value for clinicians and researchers, facilitating the assessment of leuprorelin as a viable treatment option for CPP management. Moreover, our findings contribute to filling a gap in the existing literature regarding the long-term management of CPP with leuprorelin therapy. Additionally, this systematic review and meta-analysis provides potential further direction in evaluating the long-term efficacy and safety of leuprorelin treatment.

However, our study does have some limitations. Firstly, the meta-analysis included a relatively small sample size, which may introduce bias into our findings. Although efforts were made to minimize bias through rigorous selection criteria and data extraction procedures, the limited number of studies available for inclusion may affect the robustness of our conclusions. Secondly, high heterogeneity was identified in the meta-analysis, which may be attributed to variations in patient characteristics, study methodologies, or treatment protocols at baseline. The heterogeneity in CPP diagnosis across studies poses a significant limitation. Variations in the use of clinical and biochemical parameters, as well as differing thresholds for basal and stimulated LH levels, may introduce bias. The reliance on follow-up periods to distinguish progressive from non-progressive forms further complicates comparisons. Future studies with standardized methodologies and larger sample sizes are warranted to further explore the sources of heterogeneity and validate our findings. Thirdly, the majority of children with CPP included in our analysis were girls, limiting our ability to reliably assess the impact of leuprorelin treatment in boys with CPP. Future research should strive to include a more balanced representation of both genders to better understand the efficacy and safety of leuprorelin treatment in this population. Lastly, the long-term risk of PCOS in individuals treated with GnRH agonists for CPP remains a topic of debate [30,31]. Some studies suggest an increased prevalence of PCOS in treated populations, while others find no significant difference compared to untreated individuals. Factors such as underlying predispositions, variations in diagnostic criteria for PCOS, and differences in follow-up durations contribute to these conflicting findings. Our prior study [7] found no significant difference in PCOS incidence between GnRH-treated and untreated groups. Consequently, while there is an observed association between GnRH agonist treatment and PCOS, causality cannot be definitively established.

The findings of this study have some implications for clinical practice. Central precocious puberty is characterized by the early activation of the hypothalamic–pituitary–gonadal axis before normal physiological age ranges, often accompanied by accelerated growth velocity and advancement in bone age. GnRHa therapy, which includes leuprorelin, has long been recognized as a promising approach for managing CPP. Our findings suggest that leuprorelin treatment is associated with improved adult height in children with CPP. These benefits can significantly impact the psychosocial well-being and quality of life of affected children, as well as potential long-term health outcomes. Additionally, the favorable safety profile of leuprorelin treatment, with a relatively low incidence of PCOS, further supports its use as a therapeutic option for managing CPP.

While GnRHa therapy has been shown to improve final adult height in girls with early-onset, rapidly progressive CPP, its benefit is less clear in those whose pubertal onset occurs closer to the physiological age. Some studies indicate that untreated girls with slowly progressing CPP can achieve final heights within their target range, suggesting that immediate intervention may not be necessary in all cases [32]. However, in clinical practice, physicians frequently initiate treatment for children exhibiting rapidly progressive puberty (sexual development begins after a defined age, and the process of sexual development and skeletal maturation is rapid, potentially affecting final adult height). This discrepancy between research evidence and real-world application underscores the need for further investigation to establish optimal therapeutic strategies for this patient population. Therefore, individualized treatment decisions, considering factors such as age at onset, rate of progression, and bone age advancement, are essential in managing idiopathic CPP.

In addition to leuprorelin, other GnRH agonists are utilized in the management of CPP. Another commonly utilized GnRH is Triptorelin, administered via intramuscular injection every 1, 3, or 6 months [33,34], offers the advantage of varied dosing intervals, especially the 6-month triptorelin pamoate, potentially enhancing patient compliance; however, it may be associated with injection site reactions, and further original investigations providing longitudinal efficacy and safety data are warranted, in addition to comprehensive systematic reviews [29].

In conclusion, our systematic review and meta-analysis provides comprehensive evidence supporting the long-term efficacy and safety of leuprorelin treatment in children with CPP. Despite some limitations, including small sample sizes and high heterogeneity, our findings highlight the potential benefits of leuprorelin therapy in improving adult height and menstrual regularity in this population. Future research should aim to address these limitations and further elucidate the clinical implications of leuprorelin treatment in children with CPP.

## Data Availability

Original data generated and analyzed during this systematic review are included in this article.

## Supplementary Materials

The following supporting information can be downloaded at: https://www.mdpi.com/article/10.3390/children12060712/s1, Supplementary S1, Supplementary Text: Search Strategy. Supplementary S2, Table S1: The Quality of the Studies Assessment Using the Newcastle Ottawa Scale (NOS). Supplementary S3, Table S2: Summary of Reported Data on Bone Mineral Density.

## Abbreviations

The following abbreviations are used in this manuscript:

CPP	Central precocious puberty
GnRHa	Gonadotrophin-releasing hormone analogs
LH	Luteinizing hormone
FSH	Follicle-stimulating hormone
BMI	Body mass index
NOS	Newcastle Ottawa Scale
BMD	Bone mineral density
MD	Mean difference
CIs	Confidence intervals
RR	Risk ratio
SDS	Standard deviation score
PCOS	Polycystic ovary syndrome
