# Supplementary material for: Long-Term Efficacy and Safety of Leuprorelin Treatment in Children with Central Precocious Puberty: A Systematic Review and Meta-Analysis
Source: Children (Basel). 2025 May 30;12(6):712. doi: 10.3390/children12060712 (PMC12191920; doi:10.3390/children12060712)
Supplement: Supplementary file 1 [file children-12-00712-s001.zip › children-3560839-supplementary.pdf]

Long-term efficacy and safety of Leuprorelin treatment in children with central precocious puberty: a systematic review

Online Supplementary Materials

Supplemental S1:

Supplementary Text. Search strategies

2023-11-15

- PubMed

#1. "Puberty, Precocious"[Mesh] OR Precocious Pubert*[tw] OR Pubertas Praecox*[tw] OR Sexual Precocit*[tw] OR premature pubarch*[tw] OR premature pubert*[tw] OR premature thelarch*[tw] OR proeotia*[tw] OR proiotia*[tw] OR pubertasprecox[tw] OR puberty praecox[tw] OR sex precocit*[tw] OR sexual precocit*[tw] 7159

#2. "Leuprolide"[Mesh] OR Leuprorelin[tw] OR Leuprolide[tw] OR cam2032[tw] OR daronda[tw] OR elityran[tw] OR enantone[tw] OR leuprone[tw] OR leuplin[tw] OR "la 2575"[tw] OR leuprorelina[tw] OR "lorelin depot"[tw] OR lutrate[tw] OR Lupron[tw] OR "procren depot"[tw] OR prostap[tw] OR reliser[tw] OR sixantone[tw] OR "vp 4896"[tw] 3933

#3. #1 and #2 261

#4. "Meta-Analysis"[pt] OR "Meta-Analysis as Topic"[Mesh] OR "Systematic Review" [pt] OR "Systematic Reviews as Topic"[Mesh] OR "systematic"[Filter] OR "Systematic Review*"[tiab] OR "Meta-Analysis"[tiab] OR Metaanalys*[tiab]

#5. ("controlled clinical trial"[pt] OR "Controlled Clinical Trials as Topic"[MeSH] OR "Random Allocation"[MeSH] OR "Double-Blind Method"[MeSH] OR "single-blind method"[MeSH] OR "Control Groups"[MeSH] OR "cross-over studies"[MeSH] OR random*[tiab] OR placebo[tiab] OR trial[tiab] OR groups[tiab] OR crossover[tiab] OR cross-over[tiab]) NOT ("Animals"[Mesh] NOT ("Humans"[Mesh] AND "Animals"[Mesh]))

#6. ("Observational Study"[pt] OR "Observational Studies as Topic"[Mesh] OR "Cohort Studies"[Mesh] OR "Case-Control Studies"[Mesh] OR "Cross-Sectional Studies"[Mesh] OR Observational Stud*[tiab] OR Cohort[tiab] OR "Follow-Up"[tiab] OR Longitudinal*[tiab] OR Prospectiv*[tiab] OR Retrospectiv*[tiab] OR "Case-Control"[tiab] OR "Cross-Sectional"[tiab] OR "case series"[tiab] OR "single arm"[tiab]) NOT ("Animals"[Mesh] NOT ("Humans"[Mesh] AND "Animals"[Mesh]))

#7. #3 and #4 0

#8. #3 and #5 68

#9. #3 and #6 103

- Embase

#1. 'precocious puberty'/exp OR (((Precoci* OR Praecox* OR premature) NEAR/3 (Pubert* OR Sexual OR sex)) OR (premature NEAR/3 (pubarch* OR thelarch*)) OR proeotia* OR proiotia* OR pubertasprecox):ab,ti,kw 11919

#2. 'leuprorelin'/exp OR (Leuprorelin OR Leuprolide OR cam2032 OR daronda OR elityran OR enantone OR leuprone OR leuplin OR "la 2575" OR leuprorelina OR "lorelin depot" OR lutrate OR Lupron OR "procren depot" OR prostap OR reliser OR sixantone OR "vp 4896"):ab,ti,kw 13632

#3. #1 and #2 754

#4. 'meta analysis'/exp OR 'meta analysis (topic)'/exp OR 'systematic review'/exp OR 'systematic review (topic)'/exp OR (Systemat* NEAR/3 Review* OR 'Meta Analysis*' OR Metaanalys*):ab,ti,kw

#5. ('controlled clinical trial'/exp OR 'Controlled Clinical Trial (Topic)'/exp OR 'double blind procedure'/de OR 'control group'/de OR 'crossover procedure'/de OR 'single blind procedure'/de OR 'triple blind procedure'/de OR 'placebo'/de OR 'randomization'/exp OR (random* OR trial OR groups OR placebo* OR crossover OR "cross-over"):ab,ti,kw) NOT (('nonhuman'/exp OR 'animal'/exp) NOT 'human'/exp)
#6. ('observational study'/exp OR 'cohort analysis'/exp OR 'cross-sectional study'/exp OR 'case control study'/exp OR ("observational stud*" OR Cohort OR "Follow-Up" OR Longitudinal* OR Prospectiv* OR Retrospectiv* OR "Case-Control" OR "Cross-Sectional" OR "case-series" OR "single arm"):ab,ti,kw) NOT (('nonhuman'/exp OR 'animal'/exp) NOT 'human'/exp)
#7. #3 and #4 13
#8. #3 and #5 165
#9. #3 and #6 224

- COCHRANE

#1 MeSH descriptor: [Puberty, Precocious] explode all trees 96

#2 (((Precoci* OR Praecox* OR premature) NEAR/3 (Pubert* OR Sexual OR sex)) OR (premature NEAR/3 (pubarch* OR thelarch*)) OR proeotia* OR proiotia* OR pubertasprecox):ti,ab,kw 247

#3 #1 or #2 247

#4 MeSH descriptor: [Leuprolide] explode all trees 761

#5 Leuprorelin OR Leuprolide OR cam2032 OR daronda OR elityran OR enantone OR leuprone OR leuplin OR "la 2575" OR leuprorelina OR "lorelin depot" OR lutrate OR Lupron OR "procren depot" OR prostap OR reliser OR sixantone OR "vp 4896" 1517

#6 #4 or #5 1517

#7 #3 and #6 36，all trials

Supplemental S2:

Table S1. The quality of the studies assessment using the Newcastle Ottawa Scale (NOS)

**Study ID**	**Selection**				**Comparability**	**Outcome**			**Total score**
	Representativeness of the exposed cohort	Selection of the non-exposed cohort	Ascertainment of exposure	Demonstration that outcome of interest was not present at start of study	Comparability of cohorts on the basis of the design or analysis controlled for confounders	Assessment of outcome	Was follow-up long enough for outcomes to occur	Adequacy of follow-up of cohorts	
	a) Truly representative (one star)	a) Drawn from the same community as the exposed cohort (one star)	a) Secure record (e.g., surgical record) (one star)	a) Yes (one star)	a) The study controls for age, sex and marital status (one star)	a) Independent blind assessment (one star)	a) Yes (one star)	a) Complete follow up- all subject accounted for (one star)	
	b) Drawn from a different source	b) Drawn from a different source	b) Structured interview (one star)	b) No	b) Study controls for other factors (list) ______one star)	b) Record linkage (one star)	b) No	b) Subjects lost to follow up unlikely to introduce bias- number lost less than or equal to 20% or	

															description of those lost<br>suggested no different from those followed. (one star)	
	c) Selected group		c) No description of the derivation of the non exposed cohort		c) Written self report				c) Cohorts are not comparable on the basis of the design or analysis controlled for confounders		c) Self report		Indicate the median duration of follow-up and a brief rationale for the assessment above:__________		c) Follow up rate less than 80% and no description of those lost	
	d) No description of the derivation of the cohort				d) No description						d) No description				d) No statement	
					e) Other						e) Other					
	Option	Score	Option	Score	Option	Score	Option	Score	Option	Score	Option	Score	Option	Score	Option	Score

Somchit Jaruratana sirikul 2011	a	1	a	1	a	1	a	1	a	1	d	0	a	1	b	1	7
Yi-Chun Lin 2017	a	1	a	1	a	1	a	1	a	1	d	0	a	1	b	1	7
Vickie Wu2021	a	1	a	1	a	1	a	1	a	1	d	0	a	1	b	1	7
Toshiaki Tanaka 2005	a	1	a	1	a	1	a	1	a	1	d	0	a	1	b	1	7
Sun-Jin Lee 2022	a	1	a	1	a	1	a	1	a	1	b	1	a	1	b	1	8
Shinyoung Jang 2022	a	1	a	1	a	1	a	1	a	1	d	0	a	1	b	1	7
Renata Iannetta 2015	a	1	a	1	a	1	a	1	a	1	b	1	a	1	b	1	8
Pınar Şimşek Onat 2020	a	1	a	1	a	1	a	1	a	1	b	1	a	1	b	1	8
Peter A Lee 2011	a	1	a	1	a	1	a	1	a	1	b	1	a	1	b	1	8

Maria de Fátima Borges 2015	a	1	a	1	a	1	a	1	a	1	d	0	a	1	b	1	7
Inge M. van der sluis 2002	a	1	a	1	a	1	a	1	a	1	b	1	a	1	b	1	8
Hae Sang Lee 2018	a	1	a	1	a	1	a	1	a	1	d	0	a	1	b	1	7
E. Kirk Neely 2010	a	1	a	1	a	1	a	1	a	1	b	1	a	1	b	1	8
Carolina O. Ramos 2021	a	1	a	1	a	1	a	1	a	1	b	1	a	1	b	1	8
Ah Young Cho 2020	a	1	a	1	a	1	a	1	a	1	d	0	a	1	b	1	7

Supplemental S3:

Table S2. Summary of reported data on bone mineral density

Study ID	Group	BMDf (g/cm^2)	BMDl (g/cm^2)	BMDv
Renata Iannetta 2015	Treatment group (n = 27)	0.9 ± 0.1*	0.93 ± 0.09*	0.25 ± 0.03*
	Healthy group (n = 26)	0.9 ± 0.1	0.9 ± 0.1	0.25 ± 0.03
Inge M. vander sluis 2002		LS (SD scores) (SEM)	TB (SD scores) (SEM)	
	Baseline (n = 44)	0.67 ± 0.18	0.19 ± 0.19	
	0.5 years (n = 45)	0.83 ± 0.16#	0.39 ± 0.19	
	1 years (n = 45)	0.65 ± 0.17	0.50 ± 0.17#	
	2 years (n = 39)	0.48 ± 0.18	0.38 ± 0.19	
	3 years (n = 24)	0.39 ± 0.25	0.31 ± 0.27	

*: Compared with healthy group, P value > 0.05

#: Compared with baseline, P value < 0.01

BMDl: BMD of the first to fourth lumbar vertebrae, BMDf: BMD of the total femur, BMDv: BMD of the volumetric bone density, LS: lumbar spine, SD: standard deviation, SEM: standard error of the mean, TB: total body
